# An efficient computational method for predicting drug-target interactions using weighted extreme learning machine and speed up robot features

**DOI:** 10.1186/s13040-021-00242-1

**Published:** 2021-01-20

**Authors:** Ji-Yong An, Fan-Rong Meng, Zi-Ji Yan

**Affiliations:** 1 Engineering Research Center of Mine Digitalization (China University of Mining and Technology), Ministry of Education, Xuzhou, China; 2 School of Computer Science and Technology, China University of Mining and Technology, Xuzhou, 21116 Jiangsu China

**Keywords:** DTIs, WELM, SURF, PSSM

## Abstract

**Background:**

Prediction of novel Drug–Target interactions (DTIs) plays an important role in discovering new drug candidates and finding new proteins to target. In consideration of the time-consuming and expensive of experimental methods. Therefore, it is a challenging task that how to develop efficient computational approaches for the accurate predicting potential associations between drug and target.

**Results:**

In the paper, we proposed a novel computational method called WELM-SURF based on drug fingerprints and protein evolutionary information for identifying DTIs. More specifically, for exploiting protein sequence feature, Position Specific Scoring Matrix (PSSM) is applied to capturing protein evolutionary information and Speed up robot features (SURF) is employed to extract sequence key feature from PSSM. For drug fingerprints, the chemical structure of molecular substructure fingerprints was used to represent drug as feature vector. Take account of the advantage that the Weighted Extreme Learning Machine (WELM) has short training time, good generalization ability, and most importantly ability to efficiently execute classification by optimizing the loss function of weight matrix. Therefore, the WELM classifier is used to carry out classification based on extracted features for predicting DTIs. The performance of the WELM-SURF model was evaluated by experimental validations on *enzyme*, *ion channel*, *GPCRs* and *nuclear receptor* datasets by using fivefold cross-validation test. The WELM-SURF obtained average accuracies of 93.54, 90.58, 85.43 and 77.45% on *enzyme*, *ion channels*, *GPCRs* and *nuclear receptor* dataset respectively. We also compared our performance with the Extreme Learning Machine (ELM), the state-of-the-art Support Vector Machine (SVM) on *enzyme* and *ion channel*s dataset and other exiting methods on four datasets. By comparing with experimental results, the performance of WELM-SURF is significantly better than that of ELM, SVM and other previous methods in the domain.

**Conclusion:**

The results demonstrated that the proposed WELM-SURF model is competent for predicting DTIs with high accuracy and robustness. It is anticipated that the WELM-SURF method is a useful computational tool to facilitate widely bioinformatics studies related to DTIs prediction.

## Background

The knowledge of drug-target interactions (DTIs) is much essential for drug development, so more and more studies have pay attention to identify drug-target interactions (DTIs). Identifying of novel DTIs can provide a certain help in drug development and finding new target proteins and discovering new drug candidates [1, 2]. In recent years, many experimental methods have been developed for identifying associations between drug and target protein, however, which are expensive and time-consuming. Developing a successful new chemistry-based drug usually costs billions of dollars, and it takes nearly a decade to bring the drug into market. However, only a few drug candidates are approved for marketing by Food and Drug Administration (FDA) [3–5]. The major reason is that lack of knowledge of DTIs, resulting in unacceptable toxicity for drug candidates. However, more and more studies have shown that the DTIs can provide a significant effect on the toxic side effects or toxicity of drug compounds. The knowledge of protein-target interactions can provide a certain help in finding the toxicity of drug candidates [6]. In addition, identifying interactions between protein and target can also help detecting new potential targets for an old drug and finding many potential drug candidates for a new drug target. Identifying of all potential targets could bring about a better understanding of potential toxicity and treatment of other diseases. Because of the inherent disadvantages of experimental methods, it is an urgent task for developing efficient computational approaches to identify DTIs. As a result, using computational approaches for predicting DTIs is becoming more and more important. New potential drug–target interaction candidates could be discovered by using computational methods. This make it can reduce the cost and time of experimental methods and provide a useful validation for biological experimental.

With the completion of the human genome project and the advent of molecular medicine, and with the rapid development of computer technology and biotechnology, the number of biology and chemistry biomedical literature is growing rapidly. This enables researchers to restudy the problem related to DTIs through system integration. In order to computational predict DTIs, many related databases have been established, some of which are freely available from the public sector and pay attention to relationships between drug and target, for example, Kyoto Encyclopedia of Genes and Genomes (KEGG) [7] SuperTarget and Matador [8], DrugBank [9, 10] and Therapeutic Target Database (TTD) [11, 12]. The most important help is that the data stored in these databases can provide an amount of essential experimental materials for researchers to develop new computational methods for detecting DTIs on large-scale and widely genome.

Because of the importance of identifying DTIs, a large number of computational approaches have been presented to detect DTIs. These methods can be classified as two categories: the ligand-based virtual screening approach and docking simulation. The first method compares the similarity of a given protein based on chemical structure with a classical SAR framework to predict DTIs [13]. However, this method has the disadvantage of not using protein domain information. The second method is a very useful tool of molecular modeling, which can detect the positive interactions between drug molecules and proteins by dynamically simulating the binding between drug molecules and proteins [14–16]. However, the method has a significantly disadvantage that it can be only applied to the proteins of known 3D protein structure. So far, all proteins only contain a fraction of the proteins of known 3D protein structure, therefore, the Docking simulation method is difficult to meet the experimental conditions. In addition, compared with the data of known 3D protein structure, more and more protein sequence data have been detected, and the protein sequence data are increasing exponentially. Therefore, it is very urgent research for develop efficient computational approaches based on protein sequence to identify DTIs.

Recently, a large number of computational methods have been developed to identify DTIs. Yang et al [17] proposed a computational method for finding optimal multi-objective intervention schemes in disease networks. For better recovering the disease network to the desired normal state, the method attempts to identify effective intervention points and combinations of interventions in a given disease network. Kun-Yi Hsin et al [18] proposed a new computational method, which combines two machine learning models carefully developed with multiple docking packages to evaluate the binding potential of a test compound to proteins involved in complex molecular networks. The prediction model obtained good prediction results. Francisco et al [19] presented a approach for identifying DTIs, which used molecular 2D descriptors to generate drug feature vectors. Chen et al [20] developed an effective classifier to detect DTIs by integrating the chemical-protein connections information and chemical-chemical similarities information. Yan et al [21] proposed a new feature extraction method, which used the similarity of drug chemical and target protein sequence to represent drug-target pairs. The random forest was employed to carry out prediction. Zhang *at el* [22] proposed a ensemble learning algorithm to boost performance of previous DTIs prediction methods through employing the SVM classifier to integrate the prediction results of previous methods. In spite of this, it is very important for researchers to develop efficient and robustness computational methods for improving prediction accuracy of identifying DTIs.

In the paper, we proposed a novel computational method called WELM-SURF based on drug fingerprints and protein evolutionary information for identifying DTIs. More specifically, for exploiting protein sequence feature, Position Specific Scoring Matrix (PSSM) is applied to capturing protein evolutionary information and Speed up robot features (SURF) is employed to extract sequence key feature from PSSM. For drug fingerprints, the chemical structure of molecular substructure fingerprints was used to represent drug as feature vector. Take account of the advantage that the Weighted Extreme Learning Machine (WELM) has short training time, good generalization ability, and most importantly ability to efficiently execute classification by optimizing the loss function of weight matrix. Therefore, the WELM classifier is used to carry out classification based on extracted features for predicting DTIs. The performance of the WELM-SURF model was evaluated by experimental validations on enzyme, ion channel, GPCRs and nuclear receptor datasets by using fivefold cross-validation test. The WELM-SURF obtained average accuracies of 93.54, 90.58, 85.43 and 77.45% on enzyme, ion channels, GPCRs and nuclear receptor dataset respectively. We also compared our performance with the Extreme Learning Machine (ELM), the state-of-the-art Support Vector Machine (SVM) on enzyme and ion channels dataset and other exiting methods on four datasets. By comparing with experimental results, the performance of WELM-SURF is significantly better than that of ELM, SVM and other previous methods in the domain. The results demonstrated that the proposed WELM-SURF model is competent for predicting DTIs with high accuracy and robustness. It is anticipated that the WELM-SURF method is a useful computational tool to facilitate widely bioinformatics studies related to DTIs prediction..

## Method

### Datasets

In the work, we evaluate the performance of the WELM-SURF model on four datasets: *enzymes*, *ion channels*, *GPCRs* and *nuclear receptors*. They can be downloaded from the KEGG BRITE [7], BRENDA [23], SuperTarget [8] and DrugBank [9] databases and defined as the gold standard datasets by Yamanishi [24]. The number of known drugs for *enzymes, ion channels, GPCRs* and *nuclear receptors* are 445, 210, 233 and 54 and the count of known target protein are 664, 204, 95 and 26. After carefully screening, 5127 drug-target pairs can interact with each other. There are 2926, 1476, 635, and 90 known interactions involving *enzymes*, *ion channels, GPCRs,* and *nuclear receptors*. Therefore, we constructed positive samples for each of the four datasets.

Usually, a bipartite graph was used to represent Drug-target interaction network, where each node represent drug molecules or target protein, and each edge describes true drug-target interactions valeted by biological experiments or other methods. As can be seen from the bipartite graph, the numbers of real drug-target interactions edges are small [25]. Here, we take the enzyme dataset as an example, there are 295,480 connections (445 × 664) in the corresponding bipartite graph, of which only 2926 edges are known drug-target interactions. Therefore, the possible count of negative samples (295480–2926 = 29,255) is significantly larger than the number of positive samples (2926). As a result, this will lead to a bias problem. For addressing this problem, we randomly selected the same number of negative and positive samples. Therefore, the number of negative samples for the *enzyme, ion channel, GPCRs*, and *nuclear receptor* are 2926, 1476, 635, and 90, respectively. In fact, there may be the real drug-target pairs among these negative sample sets. However, take account of the large scale of the bipartite graph, the number of true interaction pairs defined as the negative pairs is very small.

### Feature extraction

#### Drug molecules description

Recently, a number of biological experiments have indicated that drugs with similar chemical structure have similar therapeutic functions. In order to represent drugs as feature vectors, several kinds of descriptors have been designed, such as, molecular substructure fingerprints, constitutional, topological, geometrical and quantum chemical properties. In the paper, the chemical structure of molecular substructure fingerprints was used to represent the drugs as drug feature vectors [15]. Each molecular structure is translated into a fingerprint of a structural by using the molecular fingerprints method. This make it can be defined as an 881 dimensional binary vector and its corresponding bits is 1 or 0.

#### Position specific scoring matrix (PSSM)

**Fig. 1:**
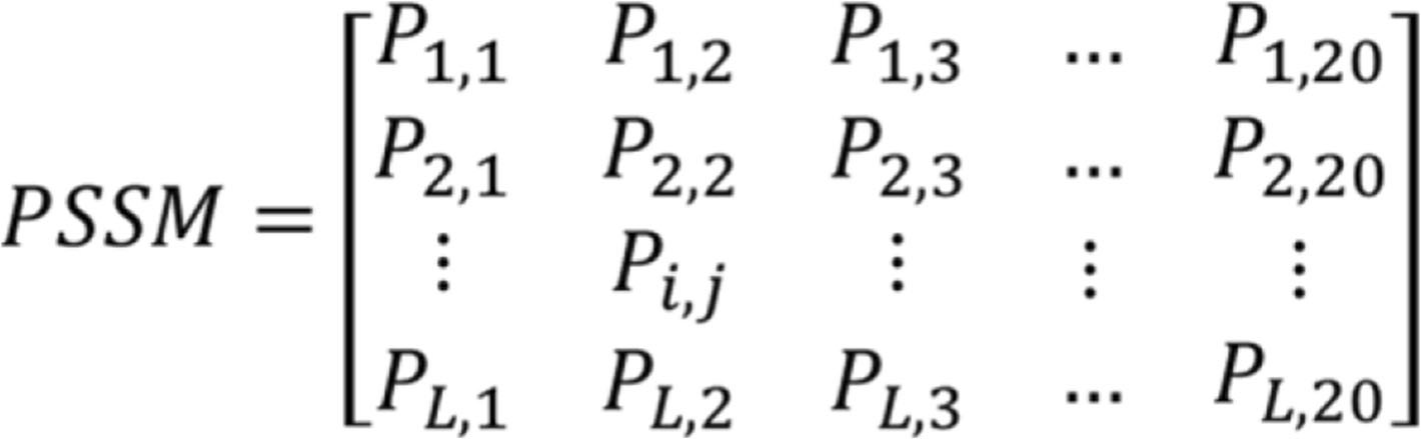
The diagram of PSSM

Due to proteins are functionally conserved, the prediction performance can be improved by using the evolutionary information of protein sequence. The position-specific scoring matrix (PSSM) contains not only the position information of the protein sequence, but also the evolution information that reflects the conservative function of protein. In the experiment, each protein sequence was converted a *L × 20* PSSM by using Position Specific Iterated BLAST (PSI-BLAST) tool [26], where *L* represents the length of different protein sequences. Therefore, we employed the PSSM for extracting the sequence evolutionary information because of its advantage in the paper. The diagram of PSSM is displayed in Fig. 1.

Where 20 are 20 different amino acids, *P*_*ij*_ represent the probability that the *i*_*th*_ amino acid in the sequence is mutated to the *j*_*th*_ type amino acid during biological evolution. The *P*_*ij*_  is greater than 0, equal to 0 and less than 0. If the *P*_*ij*_ is a positive number that indicates the *i*_*th*_ amino acid can be easily mutated to the j_th_ amino acid. In practice, the larger number of *P*_*ij*_ means a higher mutation probability. Conversely, if *P*_*ij*_ is negative number, it means the mutation probability is small, and a smaller *P*_*ij*_ number indicates more conservative. For using evolutionary information of protein sequences to capture more key features, we converted each protein sequence into a PSSM through employing PSI-BLAST tool. In the experiment, we set the parameter of PSI_BLAST’s e-value is 0.001 and selected three iterations for obtaining widely and highly homologous sequences.

#### Speed up robot features (SURF)

Speed up robot features (SURF) [27] feature extraction algorithm is an improvement of Scale Invariant Feature Transform (SIFT) algorithm [28, 29], which runs faster than SIFT in algorithm execution efficiency. The SIFT uses Gaussian differences to approximate Laplace Gauss distribution to find scale space. However, the SURF uses Box Filter to approximate LOG. The major advantage of SURF is that it is easier to calculate the convolution with the box filter by using the integrated image, which can be done in parallel at different scales. The execution of the SURF algorithm depends on the determinant of the Hessian matrix and the determinant of the position. The SURF algorithm includes the following two steps: feature point detection and feature adjacent description.

### Feature point detection

The SURF uses continuous Gaussian filters of different scales to process image and detects feature points of mesoscale invariant through Gaussian differences. SURF can represent Gaussian fuzzy approximation by using the square filter to replace the Gaussian filters of SIFT. The SURF feature extraction approach can convert a image into sets of vectors *I*_*J*_ ∈ *R*^*d*^, *j* = 1, …, *N,* where N is a number of images and *I*_*j*_ = {*f*_1_, *f*_2_, …*f*_*m*_} and ${f}_m=\left\{{f}_m^1,{f}_m^2,\dots .{f}_m^d\right\}$*,* where m is a number of local features in each image and d is the SURF features dimension that is equal to 64. The f_m_ represent the SURF local features, note that the m values are not same in all images. We want to organize *I*_*j*_ into K clusters *c* = {*c*_1_, *c*_2_, …*c*_*k*_}*.* The similarity criterion then is defined as follow equation:

$$ S\left(x,y\right)=\sum \limits_{i=1}^k\sum \limits_{j=1}^n{a}_i^j sim\left({I}_j,{m}_j\right) $$

Where $x=\left\{{a}_i^j\right\}$ *i*s separation matrix, $$ {\mathrm{a}}_{\mathrm{i}}^{\mathrm{j}}=\left\{\begin{array}{c}1,\kern0.5em \mathrm{if}\ {\mathrm{a}}_{\mathrm{i}}^{\mathrm{j}}\in \mathrm{clusters}\\ {}0,\kern0.5em \mathrm{otherwise}\end{array}\right. $$ with ${\sum}_{i=1}^k{\sum}_{j=1}^n{a}_i^j=1\forall j;y$ *= { m*_1_, …, *m*_*k*_ *}, sim*(*I*_*j*_, *m*_*j*_) represents how the correspondent features can be calculated between the two sets of local features.

The square filter can greatly improve the computation speed through using integral graph that only calculates the value the four corners of the square filter. The determinant value of hessian matrix represents the change around pixel points. Since SURF USES hessian matrix of spot detection to identify feature point whose value should be defined as the maximum or minimum value of determinant. In addition, in order to achieve scale invariance, SURF also USES the determinant of scale σ to carry out detection of feature point. For example, given a point p = (x, y) in the graph, the Hessian matrix of scale σ is can be represented as follows:

$$ H\left(p,\sigma \right)=\left(\begin{array}{c}{L}_{xx}\left(p,\sigma \right)\kern1.5em {L}_{xy}\left(p,\sigma \right)\\ {}{L}_{xy}\left(p,\sigma \right)\kern1.5em {L}_{yy}\left(p,\sigma \right)\end{array}\right) $$

Where the L_xx_(p, σ) , L_xy_(p, σ), L_xy_(p, σ) and L_yy_(p, σ) are the gray-order image after the second order differentiation. The SCALE of SURF isn’t continuous Gaussian ambiguity and down sampling processing. On the contrary, it is determined by the size of square filters. The lowest scale (initial scale) of square filter of is 9 × 9, which is approximately σ =1.2 Gaussian filter. The size of the upper scale filter will get larger and larger, such as 15 × 15, 21 × 21, 27 × 27…

The transformation formula of its scale is as follows:

$$ {\sigma}_{approx}= Currentfiltersize\times \left(\frac{BaseFilterscale}{BaseFilterSize}\right) $$

### Feature adjacent description

The descriptor of SURF uses the concept of Hal wavelet transform. In order to ensure the rotation invariance of feature point, each feature point is assigned a direction. The SURF descriptors calculate the Hal wavelet transform of 6σ pixels of direction of *X* and *Y* around feature point. A vector can be obtained by add components of corresponding *X* and *Y* of the wavelet in each interval. The longest (the largest *X* and Y components) of all vectors is the direction of the feature point. After the direction of the feature point is selected, the descriptor of feature point can be created by using the direction of surrounding pixels. For example, the 5 × 5 pixel points were defined as a sub region. As a result, a number of 16 sub regions can be generated by extracting the range of 20*20 pixel points around the feature point and the ∑*dx* and ∑ *dy* of the Hal wavelet transform in the *X* and *Y* directions within the sub region can be calculated. Finally, a feature vector with dimensional 64 can be generated.

**Fig. 2:**
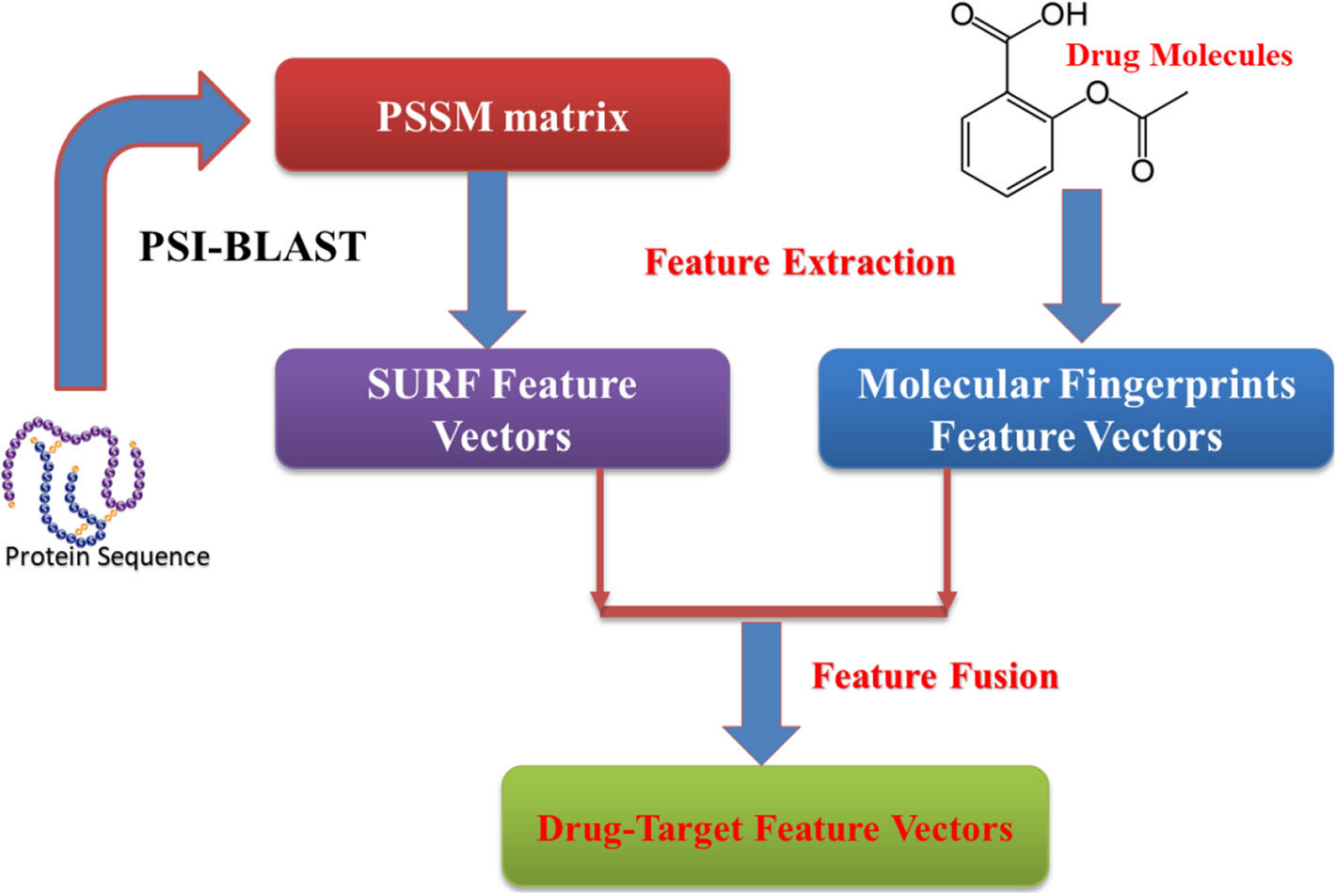
The technology roadmap of the proposed feature extraction method

In the experiment, we used SURF method to create feature vectors whose dimensional is 64. Figure 2 shows the flow diagram of our method.

### Weighted extreme learning machine (WELM)

**Fig. 3:**
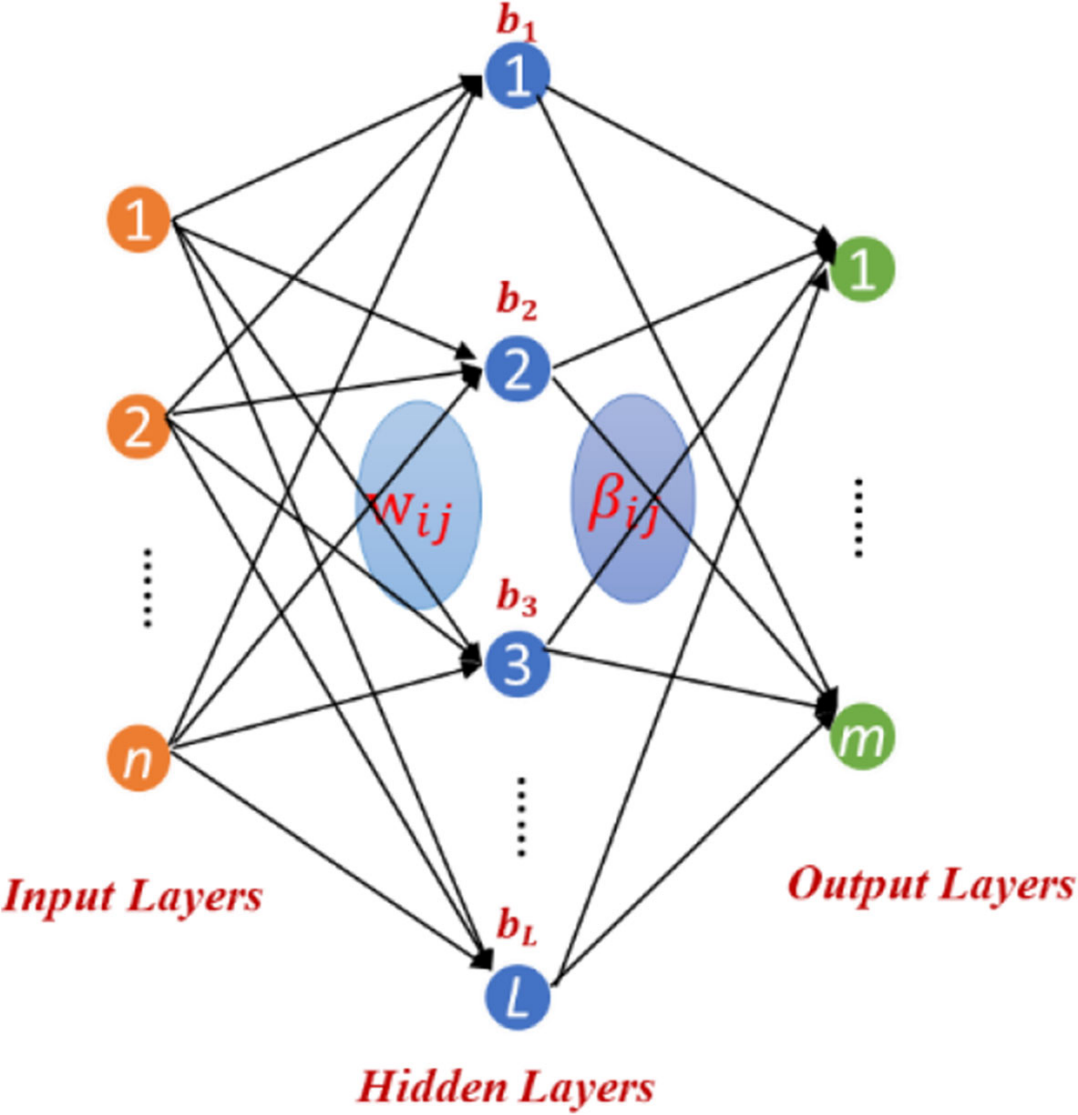
The network structure of ELM

Zong et al [30] proposed a Weighted Extreme Learning Machine (WELM) based on Extreme Learning Machine (ELM). In order to efficiently predict DTIs, we build the WELM model based on ELM. The network structure of ELM is as follows (Fig. 3):

Assuming there are *n* training samples ${\left\{{x}_i,{t}_i\right\}}_{i=1}^n$, where *x*_*i*_ = {*x*_*i*1_, *x*_*i*2_, *x*_*i*3_, ……*x*_*in*_}^*T*^ ∈ *R*^*n*^, *t*_*i*_ = {*t*_*i*1_, *t*_*i*2_, *t*_*i*3_, ……*t*_*in*_}^*T*^ ∈ *R*^*m*^, *n* represents the number of sample and *m* is the classification number. The output model of feedforward neural network with *L* hidden layer nodes can be expressed as follows:

5 $$ {\sum}_{h=1}^L{\beta}_hG\left({a}_h,{b}_h,x\right)={o}_i,i=1,2,3,\dots \dots, N $$

Where *β*_*h*_ is the output weight of the **h**_*th*_ hidden layer neuron, *G* represents activation function of hidden layer neuron, *a*_*h*_ and *b*_*h*_ is defined as the input weight and biases of hidden layer neuron, *x* is input samples, *o*_*i*_ represents the actual output value of *i*_*th*_ training sample, *t*_*i*_ is the expected output of *i*_*th*_ training sample. According to the literature [15], there are *N* training samples ${\left\{{x}_i,{t}_i\right\}}_{i=1}^n$, *x*_*i*_ ∈ *R*^*n*^. There are (*a*_*h*_, *b*_*h*_) and *β*_*h*_, which make ${\sum}_{i=1}^L\left|\left|{o}_i-{t}_i\right|\right|=0$ and single-hidden layer feedforward network (SLFN) can approach the training set ${\left\{{x}_i,{t}_i\right\}}_{i=1}^n$, *x*_*i*_ ∈ *R*^*n*^ with zero error. The eq. 1 can be simplified as follow:

6 $$ H\beta =T $$

Where *H* and *β* are the output matrix and the output weight matrix of the hidden layer respectively and *T* is the expected output matrix corresponding training samples. The output weight of the hidden layer can be expressed as follow:

7 $$ \hat{\beta}=\left\{\begin{array}{c}{H}^T{\left(\frac{I}{C}+H{H}^T\right)}^{-1}T,N<L\\ {}{\left(\frac{I}{C}+{H}^TH\right)}^{-1}{H}^TT,N\ge L\end{array}\right\} $$

The output function of ELM can be defined as follow:

8 $$ f(x)=h(x)\hat{\beta}=\left\{\begin{array}{c}h(x){H}^T{\left(\frac{I}{C}+H{H}^T\right)}^{-1}T,N<L\\ {}h(x){\left(\frac{I}{C}+{H}^TH\right)}^{-1}{H}^TT,N\ge L\end{array}\right\} $$

WELM has two weighting strategies [31], one is automatic weighting and can be defined as follow:

9 $$ {w}_1=\frac{1}{Count\left({t}_i\right)} $$

Where *Count*(*t*_*i*_) represents the number of class *t* in the training sample. The other sacrifices the classification accuracy of the majority class for obtaining the classification accuracy of the minority class. This splits the minority class and the majority class into 0.618: 1(golden ratio) and is defined as follow:

10 $$ {w}_2=\left\{\begin{array}{c}\frac{0.618}{Count\left({t}_i\right)},{t}_i\in majority\ class\\ {}\frac{1}{Count\left({t}_i\right)},{t}_i\in minority\ class\end{array}\right\} $$

The output weight of WELM hidden layer can be represented as follow:

11 $$ \hat{\beta}={H}^{-}T\left\{\begin{array}{c}{H}^T{\left(\frac{I}{C}+ WH{H}^T\right)}^{-1} WT,N<L\\ {}{\left(\frac{I}{C}+{H}^T WH\right)}^{-1}{H}^T WT,N\ge L\end{array}\right\} $$

Where the weighting matrix is a *N* × *N* diagonal matrix, and the *N* diagonal elements correspond to *N* samples. Different weights are assigned to different sample classes, and the weighting weights of the same class are the same.

**Fig. 4:**
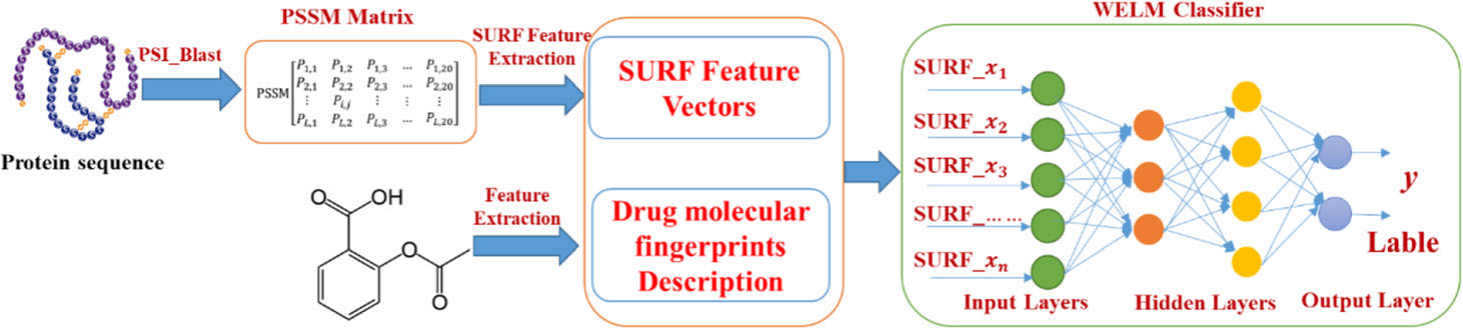
The prediction flowchart of WELM-SURF

The WELM has the advantage of short training time and good generalization ability and can efficiently execute classification by optimizing the loss function of weight matrix. As a result, the WELM classifier was used to predict DTIs by employing the automatic weighting strategy. The prediction flow diagram of WELM-SURF model is shown in Fig. 4.

### Performance evaluation

The following measures were used to evaleeuate the prediction performance of WELM-SURF in the work.

12 $$ \mathrm{Acc}=\frac{TP+ TN}{TP+ FP+ TN+ FN} $$

13 $$ \mathrm{TPR}=\frac{TP}{TP+ TN} $$

14 $$ \mathrm{PPV}=\frac{TP}{FP+ TP} $$

15 $$ \mathrm{MCC}=\frac{\left( TP\times TN\right)-\left( FP\times FN\right)}{\sqrt{\left( TP+ FN\right)\times \left( TN+ FP\right)\times \left( TP+ FP\right)\times \left( TN+ FN\right)}} $$

Where Acc represents Accuracy, TPR is Sensitivity, PPV is Precision and MCC represents Matthews’s correlation coefficient. TP and TN represent the count of real interaction and real non-interaction protein sequence pairs correctly predicted. FP and FN is the number of real non-interaction and real interaction protein sequence pairs mistakenly predicted. Meanwhile, Receiver Operating Curve (ROC) was employed to further assess the prediction performance of WELM-SURF in the work.

## Results and discussion

### Performance of the proposed method

In the experiment, we evaluate the prediction ability of our WELM-SURF model on four benchmark dataset *enzyme, ion channels, GPCRs* and *nuclear receptor*. Generally overfitting will affect experimental results. Therefore, the whole dataset was randomly divided into five parts; four parts were used as training dataset and the other part was selected as testing dataset. In addition, in order to ensure fairness, fivefold cross-validation tests was employed to evaluate the performance of the WELM-SURF and several parameters of the WELM model were optimized through using the grid search method. Here, we selected the ‘Sigmoid’ function and the ‘Gaussian ‘kernel as the mapping functions of the hidden nodes and set up Number of Hidden Neurons = 2500, C = 160 and other parameters were set up the default value. The prediction results are shown in Tables 1, 2, 3 and 4 using the WELM-SURF prediction model.

**Table 1:** Fivefold cross validation results shown using WELM-SURF method on *enzyme*

Testing set	Acc (%)	TPR (%)	PPV (%)	MCC
1	93.85	95.65	92.95	88.28
2	92.56	92.93	92.14	86.23
3	93.59	93.53	93.69	88.00
4	94.19	95.81	93.40	88.99
5	93.52	95.00	92.14	87.87
**Average**	**93.54 ± 0.61**	**94.58 ± 1.30**	**92.86 ± 0.71**	**87.89 ± 1.03**

**Table 2:** Fivefold cross validation results shown using WELM-SURF method on *ion channels*

Testing set	Acc (%)	TPR (%)	PPV (%)	MCC (%)
1	88.98	93.26	84.12	80.31
2	92.54	91.47	93.38	86.19
3	90.68	90.59	90.28	83.08
4	89.32	89.91	90.19	80.82
5	91.39	93.59	90.40	84.17
**Average**	**90.48 ± 1.47**	**91.76 ± 1.62**	**89.67 ± 3.38**	**82.91 ± 2.42**

**Table 3:** Fivefold cross validation results shown using WELM-SURF method on *GPCRs*

Testing set	Acc (%)	TPR (%)	PPV (%)	MCC (%)
1	83.07	80.95	84.30	71.84
2	88.58	86.18	89.83	79.71
3	87.40	88.00	86.81	77.97
4	84.25	85.83	83.21	73.45
5	83.86	81.34	87.20	72.88
**Average**	**85.43 ± 2.41**	**84.46 ± 3.14**	**86.23 ± 2.60**	**75.17 ± 3.46**

**Table 4:** Fivefold cross validation results shown using WELM-SURF method on *nuclear receptor*

Testing set	Acc (%)	TPR (%)	PPV (%)	MCC (%)
1	70.56	76.47	65.00	57.23
2	75.00	80.00	76.19	61.73
3	77.38	75.00	90.00	63.25
4	77.78	93.33	66.67	64.44
5	86.11	78.57	84.82	74.47
**Average**	**77.45 ± 5.67**	**80.67 ± 7.33**	**76.50 ± 10.92**	**64.22 ± 6.35**

It can be observed from Tables 1, 2, 3 and 4 that the average Accuracy for *enzymes, ion channels, GPCRs* and *nuclear receptors* is 93.54, 90.48, 85.43 and 77.45% respectively. The corresponding average Sensitivity is 94.58, 91.76, 84.46 and 80.67%, respectively. The corresponding average Precision is 92.86, 89.67, 86.23 and 76.50%, respectively. At the same time, the average Matthews’s correlation coefficient is 87.89, 82.91, 75.17 and 64.22%, respectively. These experimental results proved that good prediction performance for DTIs prediction can be achieved by using the WELM-SURF model.

The good experimental results for predicting DTIs are mainly attributed to use the SURF feature extraction method and WELM classifier. The main advantage of the WELM-SURF model is that SURF method can extract key evaluation feature from PSSM and employed chemical structure of the molecular substructure fingerprints to represent Drug feature and WELM classifier has the advantage of processing sequence data. As discussed, this is mainly due to the following three reasons: (1) The PSSM contains not only the position information of the protein sequence, but also the evolution information that reflects the conservative function of protein and a number of prior information. Therefore, it can provide a certain help in extracting evolutionary information of protein sequence. Meanwhile, the chemical structure of the molecular substructure fingerprints was use to represent Drug key feature information. (2) SURF can improve computational speed compared to SIFT. The main advantage of SURF that it uses the concept of “scale space” to capture features at multiple scale levels, which not only increases the number of available features but also makes the method highly tolerant to scale changes. This makes it can capture DTIs information and extract high efficiency features from PSSM. (3) The WELM has the advantage of short training time and good generalization ability and can efficiently execute classification by optimizing the loss function of weight matrix. Therefore, WELM is used to carry out classification and performs much better for identifying DTIs in the study. More specifically, the WELM can better perceive the distribution information of class by assigning larger weight to the minority class samples and push the separating boundary from the minority class towards the majority class through using weight strategy. This makes it can provide help in sensitive learning by assigning different weight. The results demonstrated that the proposed WELM-SURF model can improve prediction accuracy and is fit for predicting DTIs.

### Comparison with the ELM-based and SVM-based method

Despite the proposed WELM-SURF approach obtained good prediction results. However, in order to further evaluate the prediction capacity of WELM classifier, we compared its prediction ability with the ELM and the SVM by using SURF feature extraction method on *enzyme* and *ion channel* datasets. The LIBSVM tool [32] of the SVM was employed to carry out classification. At the same time, for fair comparison, several parameter of ELM were optimized through employing the same grid search method. More specifically, the number of hidden layers of ELM is set to 89 and other parameters take the default value. At the same time, the RBF kernel parameters of the SVM were optimized by using the same strategy, where c = 0.6 and g = 3.1 and other parameters were set up the default value.

**Fig. 5:**
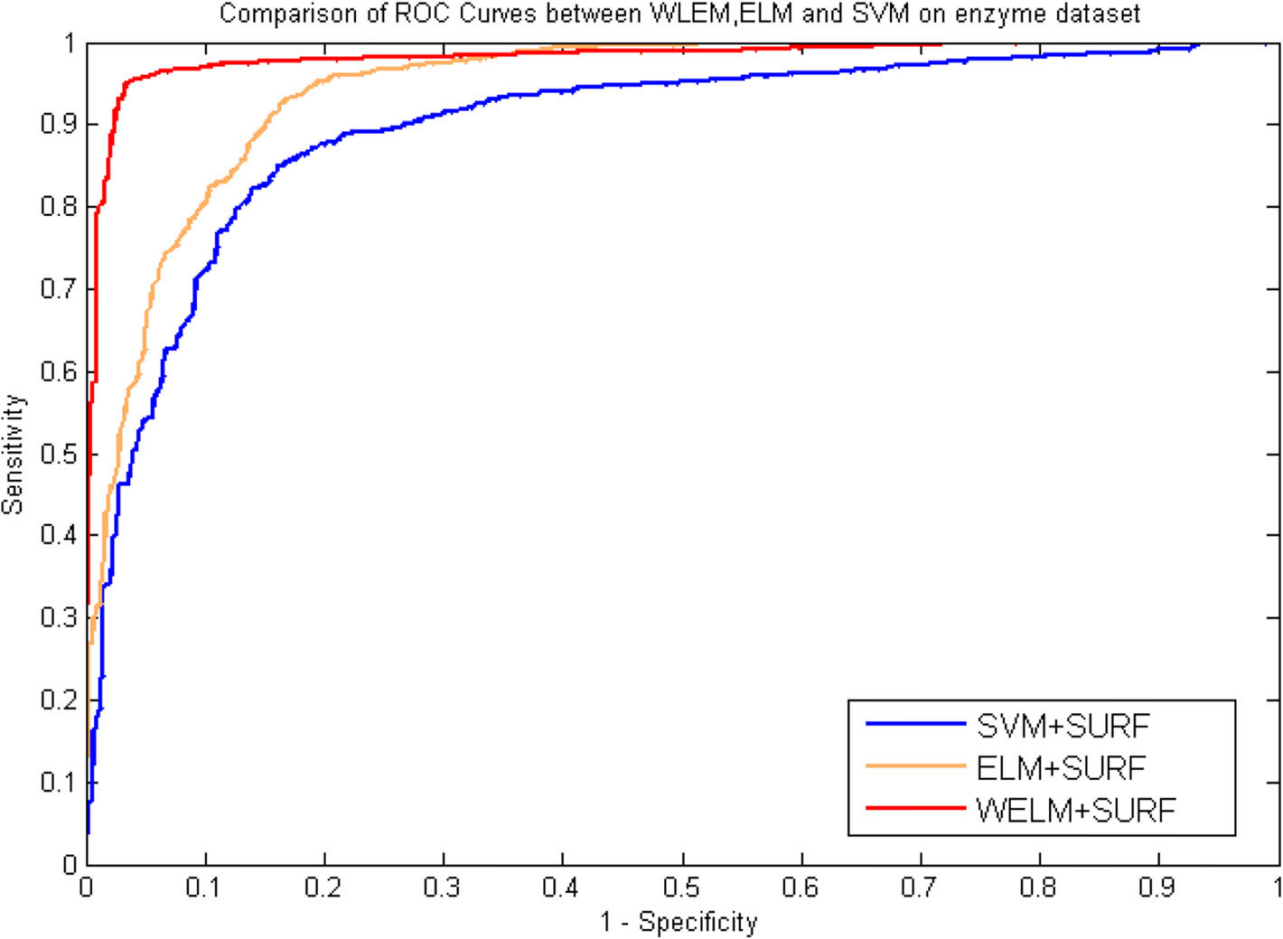
Comparison of ROC curves performed between WELM, ELM and SVM on *enzyme*

**Fig. 6:**
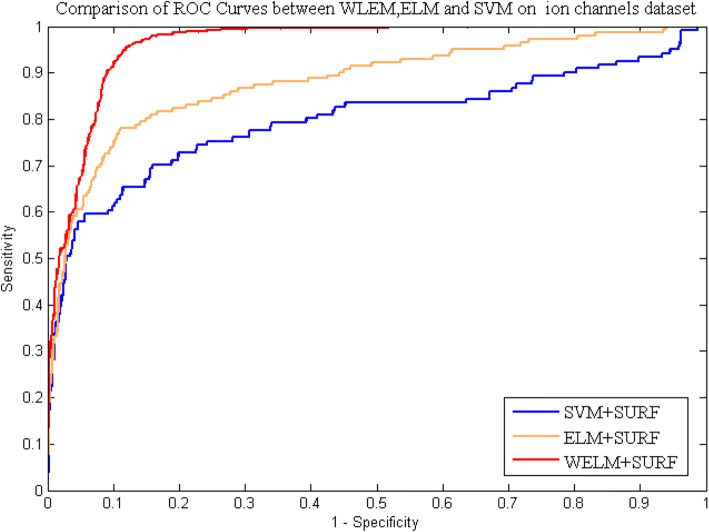
Comparison of ROC curves performed between WELM, ELM and SVM on *ion channels*

Table 5, 6, 7 and 8 list the statistical prediction results of fivefold cross-validation tests on *enzyme* and *ion channels* by using ELM classifier and SVM classifier, respectively. At the same time, the comparison of ROC Curves between WELM, ELM and SVM was also displayed in Fig. 5 and Fig. 6 on *enzyme* and *ion channels* datasets, respectively. It can be observed from Tables 5 and 6 that average accuracy of 90.38 and 87.07% obtained using ELM classifier and SVM classifier on *enzyme* dataset, while the WELM classifier achieved 93.54% average accuracy. Similarly as shown in Tables 7 and 8, 87.76% average accuracy and 83.30% average accuracy are obtained through using ELM classifier and SVM classifier on *ion channels* dataset. The WELM classifier achieved 90.48% average accuracy. It can be seen from comparison results that the prediction capacity of the WELM classifier is significantly better than that of the ELM and the SVM classifier. Similarly, we also can find from Fig. 5 and Fig. 6 that the ROC curves of the WELM classifier is also obviously better than the ELM and the SVM classifier. These good comparison results obtained may be lie in as follows reasons: The significantly advantage of WELM classifier related to the ELM classifier and the SVM Classifier is that it has the advantage of short training time and good generalization ability and can efficiently execute classification by optimizing the loss function of weight matrix, and can provide a certain help in sensitive learning by assigning different weight. Therefore, experimental results indicated that the proposed prediction model might become useful tools and can identify DTIs with a high prediction accuracy.

**Table 5:** Fivefold cross validation results shown using ELM-SURF method on *enzyme*

Testing set	Acc (%)	TPR (%)	PPV (%)	MCC (%)
1	90.75	90.81	90.57	83.20
2	89.85	88.82	90.22	81.74
3	89.58	90.37	89.19	81.33
4	90.46	90.08	90.82	82.68
5	91.26	91.23	91.22	83.55
**Average**	**90.38 ± 0.68**	**91.26 ± 0.91**	**90.38 ± 0.74**	**82.74 ± 1.27**

**Table 6:** Fivefold cross validation results shown using SVM-SURF method on *enzyme*

Testing set	Acc (%)	TPR (%)	PPV (%)	MCC (%)
1	87.78	87.30	87.14	78.50
2	87.86	85.17	89.82	78.63
3	86.84	83.48	89.58	77.09
4	85.73	83.71	88.72	75.48
5	87.12	85.34	88.24	77.53
**Average**	**87.07 ± 0.86**	**85.00 ± 1.53**	**88.70 ± 1.08**	**77.45 ± 1.28**

**Table 7:** Fivefold cross validation results shown using ELM-SURF method on *ion channels*

Testing set	Acc (%)	TPR (%)	PPV (%)	MCC (%)
1	88.51	83.32	89.75	77.98
2	87.78	82.78	88.58	76.98
3	88.16	83.78	89.65	77.95
4	87.36	81.55	89.01	78.60
5	87.06	80.07	88.40	79.21
**Average**	**87.76 ± 5.86**	**82.22 ± 1.56**	**89.08 ± 0.61**	**72.19 ± 3.05**

**Table 8:** Fivefold cross validation results shown using SVM-SURF method on *ion channels*

Testing set	Acc (%)	TPR (%)	PPV (%)	MCC (%)
1	83.56	86.89	78.91	72.40
2	82.37	84.30	80.98	70.94
3	81.19	80.84	80.56	69.43
4	82.37	82.33	84.47	70.85
5	86.99	87.50	87.78	77.31
**Average**	**83.30 ± 2.23**	**84.37 ± 2.86**	**82.54 ± 3.57**	**72.19 ± 3.05**

### Comparison with other methods

In the paper, for further evaluating the prediction capacity of WELM-SURF method, we compare our performance with four existing DIIs predictor DBSI [33], Yamanishi [24], KBMF2K [34] and NetCMP [35] on *enzyme, ion channels, GPCRs* and *nuclear receptor* dataset. These comparison results are displayed in Table 9. It can be seen from Table 9 that our prediction accuracy is obviously better than that of other four methods. The comparison results are strong evidence that the WELM-SURF is efficiently and robustness related to current exiting approaches. The results also demonstrated that the proposed WELM-SURF model is competent for predicting DTIs with high accuracy and robustness. It is anticipated that the WELM-SURF method is a useful computational tool and is suitable for predicting DTIs. The main reason is that the WELM-SURF used a good classifier and developed a novel feature extraction method.

**Table 9:** Predicting ability of different methods on *four Datasets*

Dataset	Our method	BSI [33]	Yamanishi [24]	KBMF2K [34]	NetCMP [35]
*Enzymes*	**0.9354**	0.8075	0.821	0.832	0.8251
*Icon Channels*	**0.9048**	0.8029	0.692	0.799	0.8034
*GPCRs*	**0.8543**	0.8022	0.811	0.857	0.8235
*NuclearReceptors*	**0.7745**	0.7578	0.814	0.824	0.8394

## Conclusion

In the paper, we proposed a novel computational method called WELM-SURF, which combines the Weighted Extreme Learning Machine (WELM) with Speeded up robust features (SURF) to predict DTIs based on drug fingerprints and protein evolutionary information. The WELM-SURF obtained average accuracies of 93.54, 90.58, 85.43 and 77.45% on *enzyme, ion channels, GPCRs* and *nuclear receptor* dataset respectively. We also compared our performance with the ELM classifier and the SVM classifier on *enzyme* and *ion channels* dataset and other exiting methods on four datasets. By comparing with experimental results, the performance of WELM-SURF is significantly better than that of the ELM, the SVM and other previous methods in the domain. This is mainly due to the following three reasons: (1) The PSSM contains not only the position information of the protein sequence, but also the evolution information that reflects the conservative function of protein and a number of prior information. Therefore, it can provide a certain help in extracting evolutionary information of protein sequence. Meanwhile, the chemical structure of the molecular substructure fingerprints was use to represent Drug key feature information. (2) SURF can improve computational speed compared to SIFT. The main advantage of SURF that it uses the concept of “scale space” to capture features at multiple scale levels, which not only increases the number of available features but also makes the method highly tolerant to scale changes. This makes it can capture self-protein interaction information and extract high efficiency features from PSSM. (3) The WELM has the advantage of short training time and good generalization ability and can efficiently execute classification by optimizing the loss function of weight matrix. Therefore, WELM is used to carry out classification and performs much better for identifying DTIs in the study. More specifically, the WELM can better perceive the distribution information of class by assigning larger weight to the minority class samples and push the separating boundary from the minority class towards the majority class through using weight strategy. This makes it can provide a certain help in sensitive learning by assigning different weight. We can come to the conclusion that the proposed WELM-SURF model can obtain high prediction accuracy and execute incredibly well for predicting DTIs. For the future study, more effective feature extraction approaches and machine learning algorithms can be developed for predicting DTIs.

## Data Availability

In this study, our experimental datasets can be obtained from the KEGG BRITE [7],BRENDA [23],SuperTarget [8] and DrugBank [9] databases and defined as the gold standard datasets by Yamanishi [24].

## Abbreviations

DTIs: Drug-target interactions
WELM: Weighted Extreme Learning Machine
SIFT: Scale Invariant Feature Transform
SURF: Speeded Up Robust Features
PSSM: Position Specific Scoring Matrix
ELM: Extreme Learning Machine
SVM: Support Vector Machine
PSI-BLAST: Position-Specific Iterated BLAST
Acc: Accuracy
TNR: True Negative Rate
TPR: True Positive Rate
MCC: Matthews Correlation Coefficient
PPV: Positive Predictive Value
ROC: Receiver Operating Curve
